# Novel *NBAS* mutations and fever-related recurrent acute liver failure in Chinese children: a retrospective study

**DOI:** 10.1186/s12876-017-0636-3

**Published:** 2017-06-19

**Authors:** Jia-Qi Li, Yi-Ling Qiu, Jing-Yu Gong, Li-Min Dou, Yi Lu, A. S. Knisely, Mei-Hong Zhang, Wei-Sha Luan, Jian-She Wang

**Affiliations:** 1 Department of Pediatrics, Jinshan Hospital of Fudan University, Shanghai, 201508 China; 2 The Center for Pediatric Liver Diseases, Children’s Hospital of Fudan University, Shanghai, 201102 China; 3 Institut für Pathologie, Medizinische Universität Graz, Auenbruggerplatz 25, A-8036 Graz, Austria

**Keywords:** *NBAS*, Recurrent acute liver failure, Acute liver failure, Whole exome sequencing

## Abstract

**Background:**

Underlying causes in Chinese children with recurrent acute liver failure (RALF), including liver crises less than full acute liver failure, are incompletely understood. We sought to address this by searching for genes mutated in such children.

**Methods:**

Five unrelated Chinese boys presenting between 2012 and 2015 with RALF of unexplained etiology were studied. Results of whole exome sequencing were screened for mutations in candidate genes. Mutations were verified in patients and their family members by Sanger sequencing. All 5 boys underwent liver biopsy.

**Results:**

*NBAS* was the only candidate gene mutated in more than one patient (biallelic mutations, 3 of 5 patients; 5 separate mutations). All *NBAS* mutations were novel and predictedly pathogenic (frameshift insertion mutation c.6611_6612insCA, missense mutations c.2407G > A and c.3596G > A, nonsense mutation c.586C > T, and splicing-site mutation c.5389 + 1G > T). Of these mutations, 3 lay in distal (C-terminal) regions of *NBAS,* a novel distribution.

Unlike the 2 patients without *NBAS* mutations, the 3 patients with confirmed *NBAS* mutations all suffered from a febrile illness before each episode of liver crisis (fever-related RALF), with markedly elevated alanine aminotransferase and aspartate aminotransferase activities 24-72 h after elevation of body temperature, succeeded by severe coagulopathy and mild to moderate jaundice.

**Conclusions:**

As in other countries, so too in China; *NBAS* disease is a major cause of fever-related RALF in children. The mutation spectrum of *NBAS* in Chinese children seems different from that described in other populations.

**Electronic supplementary material:**

The online version of this article (doi:10.1186/s12876-017-0636-3) contains supplementary material, which is available to authorized users.

## Background

Acute liver failure (ALF) is a rare but often fatal emergency for children, especially infants. Together with non-genetic causes such as viral infections, drug or toxin exposure, and autoimmune hepatitis, identified hereditary metabolic disorders account for half the instances of ALF in children [1–5]. Although recent work in Europe has implicated several genes in recurrent ALF (RALF) in infancy [6–10], the etiology of some instances of pediatric RALF remains unexplained. Furthermore, the causes of RALF in non-European populations are largely unexplored.

Biallelic mutations in *NBAS* (NM_015909) were first identified as causing fever-related RALF (infantile liver failure syndrome 2; MIM616483) by Haack et al. [8]. *NBAS* was previously linked to SOPH (short stature, optic nerve atrophy, and Pelger–Huët anomaly of granulocytes; MIM614800) syndrome in an isolated Russian Yakut population, but without liver failure [11]. Further observations expanded the phenotype spectrum of *NBAS* disease to involve brain, connective tissues other than bone, and the immune system as well [12–15].

Using whole-exome sequencing (WES), we evaluated 5 Chinese children with RALF. Here we describe our work and its implications.

## Methods

### Enrollment criteria

The probands were Chinese children evaluated for RALF from 2012 to 2015 by JSW, to whose clinic instances of pediatric liver disease from throughout China are referred (1096 new pediatric liver-disease patients seen during these 4y). Their parents and siblings also took part.

Participation required informed consent (for children, informed parental consent) under a protocol approved by Children’s Hospital and Jinshan Hospital of Fudan University according to the ethical guidelines of the 1975 Declaration of Helsinki. RALF of indeterminate etiology was defined as present when a child had >1 episode of liver injury, including at least 1 episode of ALF (Pediatric Acute Liver Failure Study Group criteria) [3]. That is, no child had evidence of chronic liver disease; all children had biochemical evidence of acute liver injury; all children had hepatic-based coagulopathy, with a prothrombin time (PTT) ≥15 s or an international normalized ratio (INR) >1.5 not corrected by vitamin K in the presence of hepatic encephalopathy, or a PTT ≥20s or an INR >2.0 regardless of the presence or absence of clinical hepatic encephalopathy; and all other causes possibly responsible for liver crises were excluded through comprehensive evaluation. Liver biopsy was performed when coagulopathy permitted.

### Clinical features of probands

Five unrelated boys with RALF of indeterminate etiology were enrolled in this study, including 4 of Han and 1 of Miao ancestry. The major clinical features of these probands are shown in Table 1. All parents were non-consanguine, except those of patient 4. Patient 1, initially diagnosed with liver crisis aged 6mo 18d, is the product of a 4th pregnancy (ectopic pregnancy, surgically treated; 2 induced abortions) complicated by intrahepatic cholestasis manifest as pruritus that halted after delivery. The parents are otherwise well. Patient 2, initially diagnosed in liver crisis aged 7mo 21d, is the product of a 1st pregnancy. A younger brother is well. Their mother has a 3y history of hyperthyroidism. Their father is healthy. Patient 3, initially diagnosed in ALF aged 6mo 1d, is the product of a 3rd pregnancy. A sister and brother died in fever-related liver crisis aged respectively 4mo and 8mo. Material from them suitable for genetic analysis was unavailable. Patient 4, initially diagnosed in ALF aged 6y 10mo, is the product of a 5th pregnancy (2 induced abortions, 2 live births). One sister has moyamoya disease; the other is healthy. Their parents are consanguineous. Patient 5, initially diagnosed in ALF aged 2y 2mo, is the product of a first and only pregnancy. The parents are healthy. All patients were normally grown, without dysmorphism. Patients 1–3 always had febrile illnesses before liver crises or episodes of ALF. Patient 5, with one episode of liver crisis without a febrile illness, had febrile illnesses before 3 liver crises and before one episode of ALF. No febrile illness preceded liver crisis or ALF in patient 4.

**Table 1:** Clinical features of patients 1–5

Clinical features	Patient				
	1	2	3	4	5
Ethnic group	Han	Han	Han	Miao	Han
Consanguineous parents	No	No	No	Yes	No
Birth weight (SDS)	−0.08	0.69	−0.85	NA	0.44
Age, initial RALF episode	6mo18d	7mo21d	6mo1d	6y10mo	2y2mo
Age at last assessment	6y11mo	4y8mo	2y4mo	12y11mo	4y8mo
Episodes of ALF	5	1	10	1	1
Episodes of liver crisis without ALF	3	2	1	1	4
Age at last ALF	6y11mo	2y2mo	2y4mo	6y10mo	2y2mo
Age at last liver crisis	3y1mo	4y1mo	1y7mo	9y1mo	3y10mo
Febrile illness before each episode of RALF	+	+	+	−	−
Hepatomegaly	+	+	+	+	+
Splenomegaly	+	+	+	+	+
Hepatomegaly between episodes/crises	−	−	+	NA	+
Splenomegaly between episodes/crises	−	−	−	NA	+
Body length (SDS)	−1.42	−1.47	0.31	−1.34	1.66
Age at LBX	5y11mo	2y3mo	6mo13d	9y1mo	3y10mo
Clinical status at LBX	During 7th crisis (DS)	After 2nd crisis	During 1st crisis (DS)	During 2nd crisis (DS)	During 5th crisis (DS)
ALT /AST (IU/L) at LBX	1091/307	27/12	200/24	43/22	84/48
Results of LBX	Steatosis	Centrilobular fibrosis	Steatosis; centrilobular fibrosis	Unremarkable	Inflammation
*NBAS* mutations	c.6611_6612insCA + c.3596G > A	c.3596G > A + c.586C > T	c.5389 + 1G > T + c.2407G > A	−	−

*NA* Not available, *RALF* Recurrent acute liver failure, *ALF* Acute liver failure, *ALT* Alanine transaminase (expected range 0–40 IU/L), *AST* Aspartate transaminase (expected range, 0–40 IU/L), *LBX* Liver biopsy, *DS* Downswing (resolution of crisis), *SDS* Standard deviation score. *y* Year, *mo* Month, *d* Day. All probands were male. All were born at term. None was dysmorphic. Patient 3 suffered from recurrent infections; immunologic evaluation identified no specific deficiency. Clinical-biochemistry evidence of hepatobiliary injury was seen in none between liver crises or bouts of ALF

### Liver biopsy

All 5 patients underwent percutaneous core needle liver biopsy. In patient 1, biopsy was conducted during resolution of the 4th episode of ALF (5y 11mo). Alanine transaminase (ALT) and aspartate aminotransferase (AST) values (“transaminases”) 1d before liver biopsy were abnormal (ALT/AST = 1091/307 IU/L; 0–40 expected for each). Patient 2 underwent liver biopsy 2mo after his 2nd liver crisis (2y 3mo); transaminases 2d before biopsy were normal (ALT/AST = 27/12). Patient 3 underwent liver biopsy during resolution of his 1st episode of ALF (6mo 13d). Transaminases were abnormal 4d before biopsy (ALT/AST = 200/24) and were normal 3d after biopsy (ALT/AST = 30/27). In patient 4, whose biopsy occurred during resolution of ALF (9y 1mo), ALT and AST 2d after liver biopsy were 43 and 22. Patient 5 underwent biopsy during resolution of his 5th liver crisis (3y 10mo). Transaminases 1d before liver biopsy were abnormal (ALT/AST = 84/48).

### Whole Exome sequencing

Peripheral blood samples were obtained from the probands, their parents, and their siblings. Genomic DNA was extracted routinely from peripheral blood leukocytes. Sequencing was conducted at Genesky Biotechnologies, Shanghai.

Exomes (probands; sister of patient 4) were captured using an Agilent SureSelect Human All Exon v5 kit (Agilent Technologies, Wokingham, UK). Sequencing (150 bp paired-end reads) was performed using the Illumina hiseq2500 platform following the manufacturer’s instructions (Illumina, CA). Read alignment was performed with Burrows-Wheeler Aligner (http://bio-bwa.sourceforge.net/) and Picard (https://broadinstitute.github.io/picard/). Varscan (http://varscan.sourceforge.net/) and GATK (https://software.broadinstitute.org/gatk/best-practices/) were used for variants calling. Total sequencing depth was 100X. Mean coverage of the exome ranged from 35X to 50X, with >92% of the exome covered at least 2X and >80% covered at >10X. Sequencing statistics are shown in Additional file 1.

### Exome sequencing analysis

Detailed variant filtering strategies for each patient are outlined in Additional file 2. Variants occurring with allele frequency ≥ 1% in the Thousand Genomes Project (http://www.1000genomes.org/home) and NHLBI Exome Sequencing Project (http://evs.gs.washington.edu/EVS/) databases or ≥4.5% in the Genesky in-house database were filtered out. Potential disease-causing mutations predicted by Polyphen-2 (http://genetics.bwh.harvard.edu/pph2/), SIFT (http://sift.jcvi.org/), and MutationTaster [16] (http://www.mutationtaster.org/) then were selected as suspected pathogenic variations. Suspected pathogenic variations in genes known to be associated with ALF (Additional file 3) and present in accord with inheritance modes identified candidate genes. Suspected pathogenic variations in genes not known to be associated with ALF but present in accord with recessive inheritance also identified candidate genes.

### Sanger sequencing

Polymerase chain reaction (PCR) amplification was carried out using primers specific for *NBAS* exons 8, 22, 31, 43, and 50 (Additional file 4). PCR conditions are available on request. The amplified products were sequenced using an ABI 3730xl DNA Analyzer (Applied Biosystems, Foster

City, CA) and analyzed using CodonCode Aligner software (http://www.codoncode.com). NM_015909 was used as the *NBAS* reference sequence*.*

## Results

### Identification of biallelic *NBAS* mutations in 3 RALF patients

**Fig. 1:**
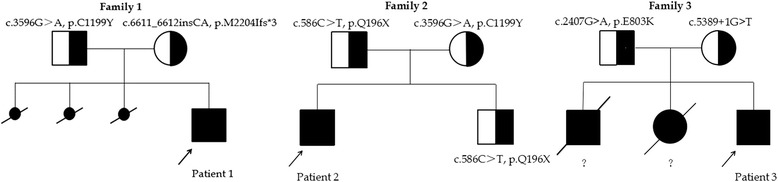
Pedigrees of families carrying two mutant alleles of *NBAS*. Blackened symbols: affected individuals.?: Liver disease in the older sister and brother of family 3 was clinically similar to that in patient 3, but no material was available to evaluate *NBAS* in either deceased older sibling

Five Chinese boys with RALF of undetermined etiology were identified from 2012 to 2015 among patients at our institutions. All underwent WES (patients 1–5), as did the healthy sister of patient 4. Filtering criteria yielded 9, 11, 13, 11, and 1 candidate genes in patients 1 through 5 respectively (Additional file 5). The only gene shared by 2 or more patients was *NBAS*. Two predictedly pathogenic variations of *NBAS* were detected in patient 1, patient 2, and patient 3, while no *NBAS* variant was detected in either patient 4 or patient 5. Sanger sequencing in parents proved compound heterozygosity in patients 1, 2, and 3 (Fig. 1).

Patient 1 harbored mutations c.3596G > A, p.C1199Y (missense) and c.6611_6612insCA, p.M2204Ifs*3 (frameshift); patient 2, mutations c.3596G > A, p.C1199Y (missense) and c.586C > T, p.Q196X (nonsense); and patient 3, c.2407G > A, p.E803K (missense) and c.5389 + 1G > T (splice-site). Two missense mutations (c.3596G > A, p. C1199Y [2 patients] and c.2407G > A, p. E803K), were both predicted to be pathogenic by Polyphen-2, SIFT, and MutationTaster [16] analyses. Patients 1 and 2 each harboured the c.3596G > A mutation. The missense mutation c.2407G > A is described (6 heterozygous instances, Exome Aggregation Consortium (ExAc) Server [http://exac.broadinstitute.org/]). The other 4 mutations are not recorded in public databases (Thousand Genomes Project; NHLBI Exome Sequencing Project; ExAc) or in the Genesky in-house database; we consider them novel.

### Clinical manifestations in 3 *NBAS* mutant patients

Unlike the 2 patients without *NBAS* mutation, those harbouring *NBAS* mutations always had a febrile illness before a liver crisis or episode of ALF. Our 3 *NBAS*-disease patients clinically resembled one another: all suffered from a febrile illness, likely infective (viral / bacterial) before each episode of RALF (fever-related RALF), with markedly elevated ALT (77–9382 IU/L; normal 0–40) and AST (213–17,344 IU/L; normal 0–40) activities 24-72 h after elevation of body temperature, succeeded by severe coagulopathy (maximum INR = 6.89; normal 0.8–1.2) and mild to moderate jaundice that were ascribed to ALF. Hypoglycemia and hepatic encephalopathy were transiently observed in patients 1 and 3. Total bile acid concentrations in all 3 patients were increased (18.2–517.2 umol/L; normal 0–10). Serum alkaline phosphatase and γ-glutamyltranspeptidase activities were normal or only mildly increased, as were blood ammonia values. Ages during episodes of ALF and liver crises are shown in Additional file 6. The details of episodes of RALF in patient 3 are shown in Additional file 7. Similar details for patients 1 and 2 are not available, as they received care at several hospitals other than ours. Biomarkers of hepatobiliary injury completely recovered between liver crises. Not all episodes of fever led to hepatic crisis. All patients used antipyretics and hepatoprotectives (e.g., ademetionine 1, 4-butanedisulfonate; reduced glutathione). In patient 1, however, during his 7th episode of RALF (4th of ALF) biomarkers returned to normal without hepatoprotectives. The 3 *NBAS*-disease patients were free from facial dysmorphism and had no broken bones. No radiogrammes were exposed in patients 1 and 2; a left lower limb radiogram in patient 3 was assessed as normal. Formal cognitive evaluation and investigations for Pelger-Huët anomaly were not conducted in any patient. Motor development was normal for all 3. Lymphocyte panels and immunoglobulin values were unremarkable in patients 1 and 3; these were not evaluated in patient 2. Ophthalmoscopy found no abnormality in patient 3 and was not conducted in patients 1 and 2.

### Liver biopsy in 5 RALF patients

Light microscopy was undertaken in patients 1–5, with ultrastructural study in patients 1–4. Hepatocyte cytoplasm contained small vacuoles in patients 1 and 3, confirmed as steatosis by transmission electron microscopy. Patients 2 and 3 had centrilobular fibrosis that was worse in patient 3. In patient 4, the liver was unremarkable. Inflammation and minimal portal-tract fibrosis were seen in patient 5. Ultrastructural findings were non-specific, with questionably increased glycogen stores in patients 1–4, dilated endoplasmic reticulum (ER) and abnormal mitochondria in patient 2, dense mitochondrial matrix in patient 3, and swollen mitochondria, questionably decreased in number, in patient 4.

## Discussion

Mutations in *NBAS* were first identified as an important cause of infantile and later-onset recurrent liver failure in 2015 [8]. NBAS is a subunit of the syntaxin 18 complex, implicated in Golgi-to-endoplasmic reticulum (ER) retrograde transport [17]. NBAS also plays an important role in nonsense-mediated mRNA decay, which regulates gene expression in response to cellular and environmental stress [18]. *NBAS* had earlier been implicated in the developmental disorder SOPH syndrome [11], in which liver disease is not a feature. And a patient with *NBAS* disease manifest as SOPH syndrome with fever-associated liver crises that fell short of RALF is described [19]. Correlations between *NBAS* mutations and clinical manifestations are incomplete.

With this report, 23 *NBAS*-disease children with recurrent liver crises are described: 14 from European countries [12, 14, 19], 3 from the United States [12, 14], 3 from Lebanon (siblings; parental consanguinity known) [13], and our 3 Han Chinese. These patients’ phenotypes range from isolated RALF to RALF in association with multisystemic disease. Our Han Chinese patients all had isolated RALF.

RALF patients usually exhibit recurrent vomiting, progressive lethargy and pyrexia 1 or 2d before medical assessment, which finds high serum transaminases; mild to moderate jaundice and severe coagulopathy then develop [8]. Our 3 *NBAS*-disease patients clinically resembled other reported children with isolated RALF [8]. All suffered from a febrile illness before each liver crisis (fever-related RALF) and at presentation had substantially elevated serum AST and ALT values, followed by mild to moderate jaundice and severe coagulopathy. However, of interest as at slight variance from published descriptions is that in our patients the frequency and severity of ALF did not lessen with increasing age (Additional file 6), and that vomiting did not usually precede rises in transaminase values.

That in *NBAS* disease raised body temperature itself might both mark and initiate a liver crisis has been suggested [14], with the corollary and experience that early and effective control of fever might prevent or alleviate liver crisis. However, in our patients peak body temperature and length of fever were not, episode for episode of RALF, positively correlated with the severity of ensuing liver crises (Additional file 7). Expression profiling has identified ER stress in cultured fibroblasts from patients with *NBAS* disease [8]. ER stress accelerates lipogenesis in the liver [20, 21] and activates the unfolded protein response, which can trigger cellular destruction through apoptosis [22, 23]. Small-vacuole steatosis in liver, although not specific for ER stress, is consistent with that etiology. Widespread loss of hepatocytes, however, as might be expected if apoptosis is triggered, was not identified even in liver biopsied during ALF with hypertransaminasemia (patient 1). Findings on microscopy did not contribute to diagnosis.

**Fig. 2:**
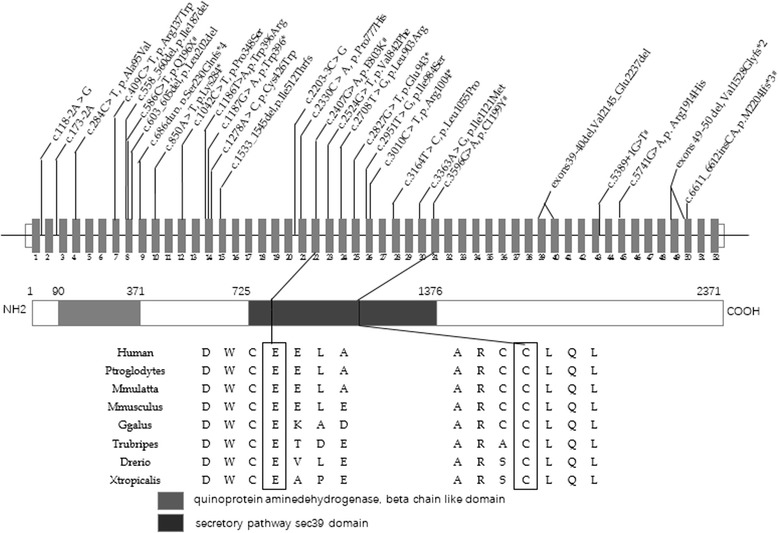
*NBAS* structure [8] and identified mutations.# Identified mutations in this study

The identified *NBAS* mutations in reported patients with liver crises comprise 5 nonsense, 14 missense, and 7 deletion/insertion mutations, with 4 splice-site variants. These are clustered into 3 regions in the first half of the gene, exons 2–4, exons 7–15 and exons 21–26 (Fig. 2). However, among the mutations identified in our patients, none of which has before been associated with RALF, 3 lie in the second half of the gene (c.6611_6612insCA, p.M2204Ifs*3; c.5389 + 1G > T; c.3596G > A, p. C1199Y, exons 50, 43, and 31 respectively). That patients 1 and 2, not identifiably related, share mutation c3596G > A, p.C1199Y may be of relevance to a special *NBAS* mutation in Han Chinese.

## Conclusions

In summary, we have identified *NBAS-*related RALF in 3 Han Chinese children. Their disorder clinically resembled that in western European (and North American, and Lebanese) children with *NBAS-*related RALF, although several of the mutations in our patients lay in a region of *NBAS* not previously found involved. RALF in each of these patients was fever-related, suggesting that to search for *NBAS* lesions in Han Chinese children with fever-related RALF may be worthwhile. That 2 of the 5 patients with RALF whom we studied – one of whom was born to consanguine parents – had no demonstrable mutations in *NBAS* or in other genes hitherto implicated in RALF implies that heritable causes of RALF remain to be discovered.

## Additional files

Additional file 1: Sequencing statistics for patients 1–5. (DOCX 14 kb)

Additional file 2: Variant filtering strategy for patients 1–5. A. Filtering procedure for suspected pathogenic genes for a single sample. B. Filtering procedure for candidate gene list. ALF, acute liver failure. (DOCX 81 kb)

Additional file 3: Reported genes associated with acute liver failure. (DOCX 16 kb)

Additional file 4: Specific primers for *NBAS* exons 8, 22, 31, 43, and 50. (DOCX 14 kb)

Additional file 5: Candidate genes for patients 1–5. (DOCX 14 kb)

Additional file 6: Ages during episodes of ALF and liver crises. P, patient. ALF, acute liver failure. N1, episodes of ALF. N2, episodes of acute liver crisis. The age of last following up for patients 1–3 is 6y 11 m, 4y 8 m, and 2y4m, respectively. With the increasing age, the episodes of ALF and RALF were not decreased (DOCX 14 kb)

Additional file 7: Details of RALF in patient 3. ALT, alanine aminotransferase; AST, aspartate aminotransferase; GGT, gamma-glutamyl transpeptidase; INR, international normalized ratio; TB, total bilirubin; TBA, total bile acids. (DOCX 15 kb)

## Abbreviations

ALF: Acute liver failure
ALT: Alanine transaminase
AST: Aspartate aminotransferase
ER: Endoplasmic reticulum
INR: International normalized ratio
PTT: Prothrombin time
RALF: Recurrent acute liver failure
SOPH: Short stature, optic nerve atrophy, and Pelger–Huët anomaly of granulocytes
WES: Whole exome sequencing
