# Supplementary material for: Novel NBAS mutations and fever-related recurrent acute liver failure in Chinese children: a retrospective study
Source: BMC Gastroenterol. 2017 Jun 19;17:77. doi: 10.1186/s12876-017-0636-3 (PMC5477288; doi:10.1186/s12876-017-0636-3)
Supplement: Supplementary file 1 — Sequencing statistics for patients 1–5. (DOCX 14 kb) [file 12876_2017_636_MOESM1_ESM.docx]

Additional file 1. Sequencing statistics for patients 1-5.

| Patient | Target Bases | | | | | Q20 | Q30 |
| --- | --- | --- | --- | --- | --- | --- | --- |
|  | ＞2X | ＞10X | ＞ 20X | ＞ 30X | Average  coverage |  |  |
| 1 | 1 | 0.976333 | 0.926959 | 0.810936 | 56.145947 | 0.938525052 | 0.859192781 |
| 2 | 1 | 0.979325 | 0.892818 | 0.823993 | 75.856502 | 0.914802762 | 0.851116018 |
| 3 | 0.996328 | 0.87772 | 0.624048 | 0.430903 | 29.126496 | 0.962624725 | 0.915629522 |
| 4 | 1 | 0.981094 | 0.896763 | 0.706882 | 49.620783 | 0.915594831 | 0.831678634 |
| 5 | 0.990615 | 0.832699 | 0.578346 | 0.381393 | 25.370375 | 0.960963584 | 0.912635339 |
| Average | 0.9973886 | 0.9294342 | 0.7837868 | 0.6308214 | 47.2240206 | 0.938502191 | 0.874050459 |
