# Supplementary material for: Novel NBAS mutations and fever-related recurrent acute liver failure in Chinese children: a retrospective study
Source: BMC Gastroenterol. 2017 Jun 19;17:77. doi: 10.1186/s12876-017-0636-3 (PMC5477288; doi:10.1186/s12876-017-0636-3)
Supplement: Supplementary file 2 — Variant filtering strategy for patients 1–5. A. Filtering procedure for suspected pathogenic genes for a single sample. B. Filtering procedure for candidate gene list. ALF, acute liver failure. (DOCX 81 kb) [file 12876_2017_636_MOESM2_ESM.docx]

Additional file 2.Variant filtering strategy for patients 1-5.


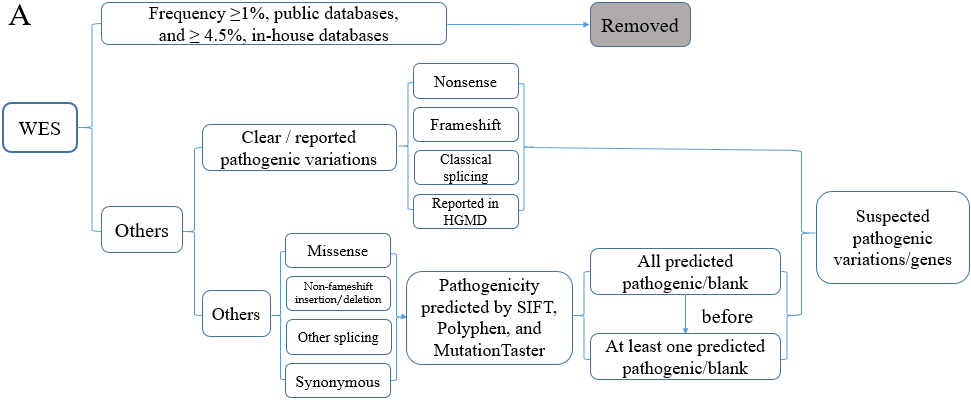


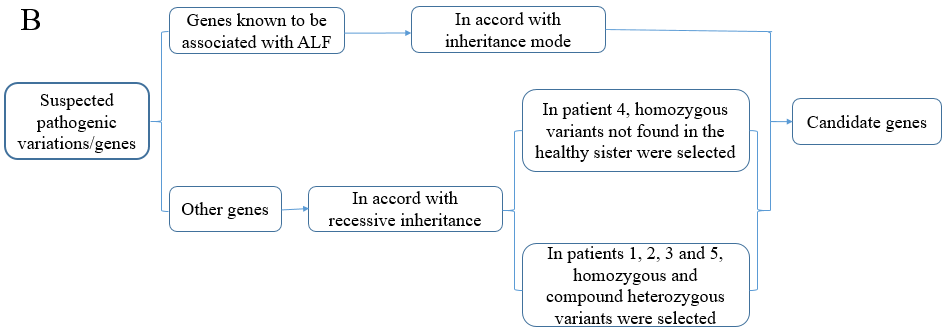


1. Filtering procedure for suspected pathogenic genes for a single sample.
2. Filtering procedure for candidate gene list.

ALF, acute liver failure
