# Supplementary material for: Novel NBAS mutations and fever-related recurrent acute liver failure in Chinese children: a retrospective study
Source: BMC Gastroenterol. 2017 Jun 19;17:77. doi: 10.1186/s12876-017-0636-3 (PMC5477288; doi:10.1186/s12876-017-0636-3)
Supplement: Supplementary file 3 — Reported genes associated with acute liver failure. (DOCX 16 kb) [file 12876_2017_636_MOESM3_ESM.docx]

Additional file 3. Reported genes associated with acute liver failure.

| Gene | Disease | Reference |
| --- | --- | --- |
| *TRMU* | Liver failure, transient infantile | 1 |
| *GFM1* | Combined oxidative phosphorylation deficiency 1 | 2 |
| *TSFM* | Combined oxidative phosphorylation deficiency 3 | 3 |
| *POLG* | Mitochondrial DNA depletion syndrome 4A (Alpers type) | 4 |
| *DGUOK* | Mitochondrial DNA depletion syndrome 3 (hepatocerebral type) | 5 |
| *MPV17* | Mitochondrial DNA depletion syndrome 6 (hepatocerebral type) | 6 |
| *LARS* | Infantile liver failure syndrome 1 | 7 |
| *NBAS* | Infantile liver failure syndrome 2 | 8 |
| *SCYL1* | Spinocerebellar ataxia, autosomal recessive 21 | 9 |
| *DLD* | Dihydrolipoamide dehydrogenase deficiency | 10 |
| *EIF2AK3* | Wolcott-Rallison syndrome | 11 |
| *ALDOB* | Fructose intolerance | 12 |

Reference

1 Zeharia A, Shaag A, Pappo O, Mager-Heckel AM, Saada A, Beinat M, et al. Acute infantile liver failure due to mutations in the *TRMU* gene. Am J Hum Genet. 2009;85:401-7.

2 Coenen MJ, Antonicka H, Ugalde C, Sasarman F, Rossi R, Heister JG, et al. Mutant mitochondrial elongation factor G1 and combined oxidative phosphorylation deficiency. N Engl J Med. 2004;351:2080-6.

3 Vedrenne V, Galmiche L, Chretien D, de Lonlay P, Munnich A, Rotig A. Mutation in the mitochondrial translation elongation factor EFTs results in severe infantile liver failure. J Hepatol. 2012;56:294-7.

4 Ferrari G, Lamantea E, Donati A, Filosto M, Briem E, Carrara F, et al. Infantile hepatocerebral syndromes associated with mutations in the mitochondrial DNA polymerase-gammaA. Brain. 2005;128:723-31.

5 Blake JC, Taanman JW, Morris AM, Gray RG, Cooper JM, McKiernan PJ, et al. Mitochondrial DNA depletion syndrome is expressed in amniotic fluid cell cultures. Am J Pathol. 1999;155:67-70.

6 Spinazzola A, Santer R, Akman OH, Tsiakas K, Schaefer H, et al. Hepatocerebral form of mitochondrial DNA depletion syndrome: novel MPV17 mutations. Arch Neurol. 2008;65:1108-13.

7 Casey JP, McGettigan P, Lynam-Lennon N, McDermott M, Regan R, Conroy J, et al. Identification of a mutation in *LARS* as a novel cause of infantile hepatopathy. Mol Genet Metab. 2012;106:351-8.

8 Haack TB, Staufner C, Kopke MG, Straub BK, Kolker S, Thiel C, et al. Biallelic Mutations in *NBAS* Cause Recurrent Acute Liver Failure with Onset in Infancy. Am J Hum Genet. 2015;97:163-9.

9 Schmidt WM, Rutledge SL, Schule R, Mayerhofer B, Zuchner S, Boltshauser E, et al. Disruptive *SCYL1* Mutations Underlie a Syndrome Characterized by Recurrent Episodes of Liver Failure, Peripheral Neuropathy, Cerebellar Atrophy, and Ataxia. Am J Hum Genet. 2015;97:855–61.

10 Brassier A, Ottolenghi C, Boutron A, Bertrand AM, Valmary-Degano S, Cervoni JP, et al. Dihydrolipoamide dehydrogenase deficiency: a still overlooked cause of recurrent acute liver failure and Reye-like syndrome. Mol Genet Metab. 2013;109:28-32.

11 Engelmann G, Meyburg J, Shahbek N, Al-Ali M, Hairetis MH, Baker AJ, et al. Recurrent acute liver failure and mitochondriopathy in a case of Wolcott-Rallison syndrome. J Inherit Metab Dis. 2008;31:540-6.

12 Muller-Wiefel DE, Steinmann B, Holm-Hadulla M, Wille L, Scharer K, Gitzelmann R. Infusion-associated kidney and liver failure in undiagnosed hereditary fructose intolerance. Dtsch Med Wochenschr. 1983;108:985-9.
