# Supplementary material for: Novel NBAS mutations and fever-related recurrent acute liver failure in Chinese children: a retrospective study
Source: BMC Gastroenterol. 2017 Jun 19;17:77. doi: 10.1186/s12876-017-0636-3 (PMC5477288; doi:10.1186/s12876-017-0636-3)
Supplement: Supplementary file 4 — Specific primers for NBAS exons 8, 22, 31, 43, and 50. (DOCX 14 kb) [file 12876_2017_636_MOESM4_ESM.docx]

Additional file 4. Specific primers for *NBAS* exons 8, 22, 31, 43, and 50.

| Exone | Forward Primer (5’ 🡺3’) | Reverse Primer (5’ 🡺3’) |
| --- | --- | --- |
| 8 | TGTACCTGAAGCCAGTGTATCAG | ATGGCTTCCAAAGCACATTT |
| 22 | ACTCCTTGTGTGGCTAAGTCA | TCAGTTCACCCATCCCACAA |
| 31 | AATGAATTCCTGGGATGTGG | ACAATGCAACAGTAAGTAGAAAACTCA |
| 43 | TCTCCCAACTGCAGGAACTT | ATCCTGGAGCAGAAGTCCAA |
| 50 | GACTTGAAGCAGCACGTCAC | TCCAGTCTCTGGGATTGCTT |
