# Supplementary material for: Novel NBAS mutations and fever-related recurrent acute liver failure in Chinese children: a retrospective study
Source: BMC Gastroenterol. 2017 Jun 19;17:77. doi: 10.1186/s12876-017-0636-3 (PMC5477288; doi:10.1186/s12876-017-0636-3)
Supplement: Supplementary file 5 — Candidate genes for patients 1–5. (DOCX 14 kb) [file 12876_2017_636_MOESM5_ESM.docx]

Additional file 5. Candidate genes for patients 1-5.

| Patients | Possible pathogenic genes | Known liver failure-causing genes |
| --- | --- | --- |
| 1 | *CSMD1, CTAGE5, DMKN, EBF4, HDC, RTFDC1, S100PBP, SASH1* | *NBAS* |
| 2 | *ABCC12, ADAMTS14, ASB18, CPA4, FRMD4A, GOLGA4, IGSF3, RAPSN, SIDT1, SLC4A3* | *NBAS* |
| 3 | *ALKBH7, ANAPC5, ATP11B, DUOX2, FAM126A, FGL1, MED16, MTMR3, PAPLN, PGRMC1, WNK3, ZRSR2* | *NBAS* |
| 4 | *APAF1, AR, ARIH1, ATRX, HGH1, MATR3, MED30, PRKG2, TMEM27, PRKD2,UXS1* |  |
| 5 | *CPS1* |  |
