# Supplementary material for: Novel NBAS mutations and fever-related recurrent acute liver failure in Chinese children: a retrospective study
Source: BMC Gastroenterol. 2017 Jun 19;17:77. doi: 10.1186/s12876-017-0636-3 (PMC5477288; doi:10.1186/s12876-017-0636-3)
Supplement: Supplementary file 6 — Ages during episodes of ALF and liver crises. P, patient. ALF, acute liver failure. N1, episodes of ALF. N2, episodes of acute liver crisis. The age of last following up for patients 1–3 is 6y 11 m, 4y 8 m, and 2y4m, respectively. With the increasing age, the episodes of ALF and RALF were not decreased (DOCX 14 kb) [file 12876_2017_636_MOESM6_ESM.docx]

Additional file 6. Ages during episodes of ALF and liver crises.

| Age | P1 | | P2 | | P3 | |
| --- | --- | --- | --- | --- | --- | --- |
|  | N1 | N2 | N1 | N2 | N1 | N2 |
| 0~1y | 0 | 1 | 0 | 1 | 4 | 0 |
| ~2y | 0 | 0 | 1 | 0 | 4 | 1 |
| ~3y | 1 | 1 | 0 | 0 | 3 | 0 |
| ~4y | 0 | 1 | 0 | 0 | - | - |
| ~5y | 2 | 0 | 0 | 1 | - | - |
| ~6y | 1 | 0 | - | - | - | - |
| ~7y | 1 | 0 | - | - | - | - |

P, patient. ALF, acute liver failure. N1, episodes of ALF. N2, episodes of acute liver crisis. The age of last following up for patients 1-3 is 6y 11m, 4y 8m, and 2y4m, respectively. With the increasing age, the episodes of ALF and RALF were not decreased.
