# Supplementary material for: Novel NBAS mutations and fever-related recurrent acute liver failure in Chinese children: a retrospective study
Source: BMC Gastroenterol. 2017 Jun 19;17:77. doi: 10.1186/s12876-017-0636-3 (PMC5477288; doi:10.1186/s12876-017-0636-3)
Supplement: Supplementary file 7 — Details of RALF in patient 3. ALT, alanine aminotransferase; AST, aspartate aminotransferase; GGT, gamma-glutamyl transpeptidase; INR, international normalized ratio; TB, total bilirubin; TBA, total bile acids. (DOCX 15 kb) [file 12876_2017_636_MOESM7_ESM.docx]

Additional file 7. Details of RALF in patient 3.

| Episode of RALF | Peak  temperature (°C) | Length of fever (days) | Maximum ALT (IU/L) | Maximum AST (IU/L) | Maximum INR | Length (days) of abnormal INR | Maximum TB (umol/L) | Maximum  GGT  (IU/L) | Maximum TBA (umol/L) | Length of hospital stay (days) |
| --- | --- | --- | --- | --- | --- | --- | --- | --- | --- | --- |
| 1 | 39.2 | 8 | 4301 | 5880 | 6.89 | 7 | 72.8 | 68 | 358.8 | 15 |
| 2 | 38.5 | 3 | 2766 | 3871 | 4.95 | 4 | 42 | 181 | 148.8 | 18 |
| 3 | 39.3 | 2 | 2269 | 1760 | 2.29 | 2 | 29.9 | 49 | 181 | 6 |
| 4 | 38.2 | 3 | 1629 | 2351 | 2.72 | 2 | 46.4 | 105 | 216.7 | 9 |
| 5 | 39.8 | 3 | 2349 | 2673 | 4.73 | 5 | 66.5 | 39 | 393.6 | 11 |
| 6 | 39.5 | 12 | 2090 | 3308 | 1.82 | 5 | 37.3 | 185 | 18.2 | 12 |
| 7 | 39.9 | 10 | 1764 | 2436 | 4.24 | 4 | 60.1 | 173 | 372.1 | 11 |
| 8 | 39.6 | 4 | 2063 | 4652 | 8.06 | 5 | 24.5 | 78 | 394 | 6 |
| 9 | 38.5 | 1 | 1478 | 2791 | 2.05 | 2 | 40.2 | 88 | 221.8 | 4 |
| 10 | 38.9 | 3 | 2993 | 4586 | 2.64 | 3 | 30.4 | 106 | 391.3 | 6 |
| 11 | 39.7 | 3 | 1309 | 2199 | 2.36 | 2 | 51.1 | 82 | 307.8 | 5 |
| Reference range | < 37.3 | 0 | 0-40 | 0-40 | 0.8-1.2 | 0 | 5.1-17.1 | 7-50 | 0-10 | 0 |

ALT, alanine aminotransferase; AST, aspartate aminotransferase; GGT, gamma-glutamyl transpeptidase; INR, international normalized ratio; TB, total bilirubin; TBA, total bile acids.
